# Supplementary material for: Agreement and relationship between measures of absolute and relative intensity during walking: A systematic review with meta-regression
Source: PLoS One. 2022 Nov 3;17(11):e0277031. doi: 10.1371/journal.pone.0277031 (PMC9632890; doi:10.1371/journal.pone.0277031)
Supplement: S1 Table — (PDF) [file pone.0277031.s002.pdf]

	Question	Operational definition	Scoring system
1	Is the hypothesis/ aim/ objective of the study clearly described?	Were all three provided, report accordingly in qualitative analysis. Point given if 1 of 3 are clearly described.	1 – Yes<br>0 – No
2	Are the main outcomes to be measured clearly described in the introduction or Methods section?	Do they clearly state the primary and secondary outcomes.	1 – Yes<br>0 – No
3	Are the characteristics of the participants included in the study clearly described?	Means and SD reported for participants characteristics.	1 – Yes<br>0 – No
4	Are the main findings of the study clearly described?	Are summary data clearly presented in table format? Is there a textual description of the main findings.	1 – Yes<br>0 – No
5	Does the study provide estimates of the random variability in the data for the main outcomes?		1 – Yes<br>0 – No
6	Have actual probability values been reported (e.g. 0.035 rather than <0.05) for the main outcomes except where the probability value is less than 0.001?	0.035 rather than <0.05 for the main outcomes except where the probability value is less than 0.001?<br>All P values must meet the criteria consistently.	1 – Yes<br>0 – No
7	Were the participants asked to participate in the study representative of the entire population from which they were recruited?	Relates to initial recruitment not participation. Have they accurately identified the population (title, intro, methods).	1 – Yes<br>0 – No
8	Were those participants who were prepared to participate representative of the entire population from which they were recruited?	Do the participants who participated closely correspond to the population identified (title, intro, methods).	1 – Yes<br>0 – No

9	Were the hypotheses of the study pre-registered? (and a link provided to the registration)	Should be reported if completed	1 – Yes<br>0 – No
10	Were the statistical tests used to assess the main outcomes appropriate?	The statistical techniques used must be appropriate to the data. For example, nonparametric methods should be used for small sample sizes. Where little statistical analysis has been undertaken but where there is no evidence of bias, the question should be answered yes. If the distribution of the data (normal or not) is not described it must be assumed that the estimates used were appropriate and the question should be answered yes.	1 – Yes<br>0 – No
11	Were the validity of the main outcome measures reported and/ or established?	Needs to be reported, referenced, with a valid and reliable method	1 – Authors have reported a valid accurate and reliable dependent variable, with verification of accuracy, reliability and validity.<br>0 - If they are reported but not accurate<br>0 - If they are not reported – unable to determine
12	Was there adequate adjustment for confounding in the analyses from which the main findings were drawn?		1 – If adjustment wasn't required<br>1 – if adjustment was required and completed<br>0 – if adjustment was required but was not completed
13	Did the study perform a power calculation, report associated inputs and recruit the required number of participants?		1 - Apriori power calculation was completed, no associated inputs recorded, did not recruit the required number of participants

2 - Apriori calculation has been completed, no inputs have been reported, successfully recruited the required participants
3 - Apriori calculation was completed with associated inputs reported, but didn’t recruit required participant numbers
4 - Apriori power calculation was completed with associated inputs reported, and recruited required participants
