# Supplementary material for: Agreement and relationship between measures of absolute and relative intensity during walking: A systematic review with meta-regression
Source: PLoS One. 2022 Nov 3;17(11):e0277031. doi: 10.1371/journal.pone.0277031 (PMC9632890; doi:10.1371/journal.pone.0277031)
Supplement: S2 Table — (DOCX) [file pone.0277031.s003.docx]

|  | **Reporting** | | | | | | **External validity** | | **Internal validity** | | | | **Power** |  |
| --- | --- | --- | --- | --- | --- | --- | --- | --- | --- | --- | --- | --- | --- | --- |
|  |  |  |  |  |  |  |  |  | **Bias** | | | **Confounding** |  |  |
|  | **1** | **2** | **3** | **4** | **5** | **6** | **7** | **8** | **9** | **10** | **11** | **12** | **13** | **Total** |
| **Agiovlasitis 2014** | 1 | 1 | 1 | 1 | 1 | 0 | 0 | 1 | 0 | 1 | 1 | 1 | 0 | 9 |
| **Tumiati 2008** | 1 | 1 | 1 | 1 | 1 | 0 | 1 | 0 | 1 | 1 | 1 | 1 | 0 | 10 |
| **Brooks 2005** | 1 | 1 | 1 | 1 | 1 | 1 | 0 | 1 | 0 | 1 | 1 | 1 | 0 | 10 |
| **Caballero 2019** | 1 | 1 | 1 | 1 | 1 | 1 | 0 | 1 | 0 | 1 | 1 | 1 | 0 | 10 |
| **Dos Anjos 2011** | 1 | 0 | 1 | 1 | 1 | 1 | 0 | 0 | 1 | 0 | 0 | 1 | 0 | 7 |
| **Ham 2007** | 1 | 1 | 1 | 1 | 1 | 1 | 0 | 1 | 0 | 1 | 1 | 1 | 0 | 11 |
| **Kilpatrick 2009** | 1 | 1 | 1 | 1 | 1 | 0 | 1 | 0 | 0 | 1 | 1 | 1 | 0 | 9 |
| **Nakanishi 2018** | 1 | 1 | 1 | 1 | 1 | 1 | 0 | 1 | 0 | 1 | 1 | 1 | 0 | 9 |
| **Ozemek 2013** | 1 | 1 | 1 | 1 | 1 | 0 | 1 | 1 | 0 | 1 | 1 | 1 | 0 | 10 |
| **Sell 2011** | 1 | 1 | 1 | 1 | 1 | 0 | 1 | 1 | 0 | 1 | 1 | 1 | 0 | 12 |
| **Gil-Rey 2018** | 1 | 1 | 1 | 1 | 1 | 0 | 1 | 1 | 0 | 1 | 1 | 1 | 0 | 10 |
| **Gil-Rey 2019** | 1 | 1 | 1 | 1 | 1 | 1 | 0 | 1 | 0 | 1 | 1 | 1 | 0 | 10 |
| **Hagins 2007** | 1 | 1 | 1 | 1 | 1 | 1 | 0 | 1 | 0 | 1 | 1 | 1 | 0 | 10 |
| **Spelman 1993** | 1 | 1 | 1 | 1 | 1 | 0 | 1 | 1 | 0 | 1 | 1 | 1 | 0 | 10 |
| **Sweeger 2020** | 1 | 1 | 1 | 1 | 1 | 1 | 1 | 1 | 0 | 1 | 1 | 1 | 0 | 11 |
