# Supplementary material for: Agreement and relationship between measures of absolute and relative intensity during walking: A systematic review with meta-regression
Source: PLoS One. 2022 Nov 3;17(11):e0277031. doi: 10.1371/journal.pone.0277031 (PMC9632890; doi:10.1371/journal.pone.0277031)
Supplement: Attachment — Submitted filename: response to reviewers comments.docx [file pone.0277031.s005.docx]

**Agreement and relationship between measures of absolute and relative intensity during walking: a systematic review with meta-regression**

**PONE-D-22-05506**

| Reviewer 1 comments | Response to Reviewer Comments |
| --- | --- |
| Surely, however, this is something that has been thoroughly discussed in the past. See for example Norton et al. (2010, JSAMS) and Iannetta et al. (2021, Sports Medicine) – on a side note, it is surprising that these references were not included in this manuscript. | Previous research has been completed in this area; however, we do not believe a systematic review with meta regression has. We acknowledge the suggested references and the Ianetta et al. 2021 study has been added (line 161 – 166) |
| Another point that should be considered is that the authors assumed that maximum-derived percent indices of intensity (e.g., VO2max, HRmax) are the benchmark metrics against which METs levels should be compared. It is more and more evident that these metrics, although potentially improving the prescription of exercise compared to a METs-based approach, are still unable to capture the true metabolic stress of any physical activity (see Iannetta et al. 2020, MSSE). What the results of this meta-analysis demonstrate is a substantial, intrinsic incoherence between the METs-derived classification and relative metrics of intensity that are deemed to characterize those categories.  Although I have no doubt that this incoherence has important practical implications, these results should not encourage the authors to imply that maximum-derived percent indices of intensity should be the “gold-standard” approach. In fairness, these limitations should also be discussed. | While we acknowledge that the relative measures used within this paper are not gold standard measures, we do not state these relative measures to be gold standard. Currently, wearable devices are limited to measuring heart rate (and not ventilatory or lactate thresholds) and thus at a population level offer the most accurate method of relative exercise intensity. To clarify this, the following has been added in lines 161-166:  ‘Yet there are some limitations with this method when compared to other forms of relative intensity such as lactate and ventilatory thresholds that do not use a fixed percentage value for exercise intensity boundaries (1). However, wearable devices do not currently have the capacity to measure such physiological thresholds and currently, %HRR may offer the most accurate relative measure available at a population level.’  This has also been referred to on lines 495 – 504  In the past, absolute intensity was preferred probably because the technology for measuring relative intensity was either not available or inappropriate. However, the expanding use of wearable devices by the wider population and the seismic growth in the wearable market (85) now offer the ability to use relative measures of intensity more easily. Evidence suggests that heart rate measured at rest and during walking by photoplethysmography sensors embedded in wearable devices is very accurate (86–88). Individualised physical activity relative to cardiorespiratory fitness is now more readily available, due to wearable devices, and therefore, physical activity and prescription should adopt relative intensity measures as part of population-based guidelines. |
| Finally, in several parts of the manuscript the authors give to the reader the impression that the METs system is often used to prescribe exercise intensity. In fact, what happens in most research and applied settings is that METs categories are used to retrospectively quantify/characterize the “training load” of physical activity, whereas they are rarely used to “prospectively” prescribe exercise intensity. This, I think, is an important distinction that the authors should consider implementing. | This was not our intention. Our intention was to describe physical activity monitoring. The manuscript has been amended to reflect this. On line 464 and 486 we refer to exercise monitoring and on lines 504 and 513 to ‘prescription and monitoring’. We believe there is importance in physical activity guidelines being prescribed as accurately as possible and thus refute the notion that exercise prescription should not use relative formats, especially when wearable devices make such information readily accessible. |
| Specific comments: The intro is quite long considering the objective of this meta-analysis. The authors could simply justify the need for this analysis based on several previous indications that the METs classification should be re-evaluated. | We have adjusted the introduction to reflect this whilst adding additional references to justify the rationale. |
| Only 15 studies included. This is not the fault of the authors, but I wonder whether the same research question could have been answered more accurately with a more empirical methodology (actual collection of data). | We agree that more empirical studies in this area are needed, but the purpose of a systematic review is to synthesise the findings of studies currently available in order to then be able to design appropriate methodology for future studies. A single study is not likely to have the sample size required to do as proposed. Moreover, the meta-regression approach has allowed us to examine the relationship between METs and %HRR in a large sample that would have been very difficult to achieve with a single study. |
| Discussion As for the intro, the discussion is quite long and, in some points, quite repetitive. I would suggest the authors to be more straight forward to avoid these repetitions. | As suggested, we have re-evaluated the discussion and removed text accordingly so that it is more succinct. |

| Reviewer 2 comments |  |
| --- | --- |
| I do not appreciate the utterly empirical approach and the lack of the physiological background that forms the very bases of the intensity prescription scheme. The authors are advised to broaden their focus to include recent pertinent work by Caen K and Teso M. for a physiological description of the concept of training intensity, the difference between absolute and relative intensity and why both these variables are important. Moreover, the authors should consider several recent papers from Iannetta D. These papers offer a physiology-based and data-driven critique of the fixed METs prescription scheme. | Whilst we acknowledge the empirical approach, physiological mechanisms were not the focus of this systematic review with meta-regression. We acknowledge the Ianetta et al. 2021 paper recommended and have added this to the introduction: line 161-166.  We have also highlighted specifically why we chose to utilise the relative measures due to their population level accessibility on lines 496 – 505  In the past, absolute intensity was preferred probably because the technology for measuring relative intensity was either not available or inappropriate. However, the expanding use of wearable devices by the wider population and the seismic growth in the wearable market (85) now offer the ability to use relative measures of intensity more easily. Evidence suggests that heart rate measured at rest and during walking by photoplethysmography sensors embedded in wearable devices is very accurate (86–88). Individualised physical activity relative to cardiorespiratory fitness is now more readily available, due to wearable devices, and therefore, physical activity and prescription should adopt relative intensity measures as part of population-based guidelines. |
| Walking is only mentioned in introduction while the authors don’t come back to it in the rest of the manuscript. Actually, the choice of restricting the focus of the paper on this form of exercise only seems unfortunate. When one considers the relatively low cost of walking locomotion (unless it is conducted on a steep slope or while carrying heavy loads) it is hard to generate anything but a moderate intensity of exercise. The validity of the study would have been higher if a wider range of absolute and relative intensities had been considered. Please discuss. | While we acknowledge other modalities of physical activity exist, we specifically chose walking as we are interested in the largest population level effect, of which walking is the most common form of physical activity performed. This is stated in the introduction:  “Walking is a very popular form of physical activity at a population level (1). It is low cost, accessible, and well-tolerated across age groups (2). Given this popularity, walking is a key intervention for physical activity promotion (3). Advocating walking (and physical activity in general) is important because it is well documented that physical activity reduces the risk of developing a range of chronic diseases (4,5).”  In the discussion we also reference walking on lines 390 and 501  Although we acknowledge that walking usually generates moderate intensity, this is the aim of the paper, to understand how walking intensity is measured during and the relationship between relative and absolute intensity. At a population level physical activity is not adhered to well, and we believe that the largest effect on public health would be to encourage sedentary populations into doing more moderate intensity physical activity. Hence the advocation of moderate relative intensity as per the physical activity guidelines and the use of wearable devices within this paper. |
| I am surprised of the relatively high absolute intensities reported in the study (>6 METs) as well as the high relative intensities (>60 %HRR). It is generally very hard to generate these absolute and relative intensities while walking. Please discuss. | Although we acknowledge walking generally elicits moderate intensity, it is possible, depending on the cardiorespiratory fitness of the individual, to achieve vigorous intensity during flat, non-loaded walking conditions. Additionally, the aim of the paper was to identify any differences between absolute and relative intensities at moderate intensity, in line with the minimum requirement intensity often prescribed by government guidelines. (England, Australia, Canada, USA all advocate for moderate intensity as the minimum intensity in exercise guidelines). |
| The above leads to another question: were METs directly measured in the studies (based on measures of VO2) or were they guidelines-based estimates? This is very important, and it should be clarified. If VO2 was not measured yours would be a rather circular argument. Please discuss. | This has been discussed in the manuscript - please refer to the section entitled “METS’ line 428 – 446 for clarity. |
| To establish the agreement between indexes of absolute and relative intensity, my understanding is that you used the ACSM’s fixed classification scheme. However, the validity of this approach has been recently questioned (Sports Med. 2021 Nov;51(11):2411-2421; Med Sci Sports Exerc. 2020 Feb;52(2):466-473). This scheme is based on incorrect assumptions: i) the relative intensity is a linear function of absolute intensity, while clearly it is not since intensity domains are defined by threshold boundaries; ii) one size fits all in terms of mutual correspondence between absolute and relative intensity, which is clearly not the case due to differences in fitness and due to the higher than expected variability in the relative position of intensity boundaries. In the above papers a more accurate and fitness-dependant mutual translation scheme between relative and absolute intensity can be found. Please discuss all of the above. | Although we acknowledge this, the ACSM suggest %HRR is a gauge of relative intensity in line with METs, %VO_2max,_ %VO_2_R etc, and currently is used as a one size fits all approach at a population level to prescribe and monitor physical activity intensity. While we appreciate and have acknowledged that other potentially more accurate methods of relative intensity monitoring exist (line 162 – 166), there is currently no feasible method of measuring/monitoring these physiological thresholds easily at a consumer/population level, hence our focus on variables that can easily be obtained from currently available wearable devices. |
| I don’t agree with your conclusion that since there is a low correspondence between absolute and relative intensity (as classified by the ACSM’s translation scheme), we should only focus on relative intensity when prescribing exercise. I think that you study, using an alternative approach compared to the physiological studies, is further corroborating the idea that the current mutual translation scheme between absolute and relative intensity should be substantially revised. However, this does not take away the importance of absolute intensity as an index of absolute energy expenditure, absolute heat production, absolute carbohydrates and fats oxidation, i.e. the “extensive” stress for the body that is generated by physical exertion. This is just as relevant towards certain health related outcomes of the “exercise pill” as the “intensive” stress that is related to the disturbance of the intracellular homeostasis in myocytes, that in turn is related to relative intensity. Absolute and relative load are both “active principles” of the exercise pill that stress the body in a different way, causing different structural and functional adaptations over time (please see J Physiol 2017 May 1;595(9):2915-2930 and successive work from the same group for a general framework). | We have not made this conclusion, nor are we questioning the importance of absolute forms of energy expenditure, heat production or fat and carbohydrate oxidation. We conclude that measures of exercise prescription and monitoring should be more highly prioritised to improve access at a population-level, not lab-based.  ‘As such, measurement of relative intensity should be more highly prioritised as part of physical activity programmes and guidelines’….. ‘to allow the wider population access to relative individualised intensity thresholds.’  Lines 514– 517. |
