# Supplementary material for: Agreement and relationship between measures of absolute and relative intensity during walking: A systematic review with meta-regression
Source: PLoS One. 2022 Nov 3;17(11):e0277031. doi: 10.1371/journal.pone.0277031 (PMC9632890; doi:10.1371/journal.pone.0277031)
Supplement: Attachment — Submitted filename: 2nd review addressing reviewer comments.docx [file pone.0277031.s006.docx]

**Response to Reviewer Comments:**

Given we have deleted sections of text, the line numbers are no longer continuous. However, the line numbers we refer to in the table below are accurate.

Thank you to both reviewers for your feedback. We have tried to accommodate your responses as best as possible.

|  | **Comments 2^nd^ review** | **Response** | **Action** |
| --- | --- | --- | --- |
| **Reviewer 1** | Comment from reviewer | Authors response | Section and page additions to manuscript |
| **1** | Reviewer #1: General comments: I want to begin this second round of review acknowledging that I think the paper is well written and it addresses a valid research question. It is unfortunate, however, that some, if not most, of my previous comments (as well as some valid ones from reviewer #2) were not taken into consideration. Also, some changes (for example those presumably made to shorten the intro and discussion) are not explained in the responses nor tracked within the updated manuscript version. | We have tried to make it clearer in this round of reviews where we have removed text from the Introduction. Please see the tracked changes document and actions taken. | Discussion section softened at lines 524 - 526 to reduce advocacy of relative intensity over absolute intensity and better reflect our intent.  Intro has been reduced from 3.5 to 2.5 pages.  Wearable device market has been moved from Intro to Discussion lines 529 – 531. |
| **2** | I’m still of the opinion that the authors are trying to “overinterpret” their data. Yes, there is a discrepancy between METs categories and relative intensity ones. Yet, I think the authors should be careful in overemphasizing this discrepancy and dismiss the use of METs. Afterall, from such a metanalytic approach one cannot derive whether this discrepancy is potentially detrimental for the characterization of exercise intensity (considering the limitations inherent to current relative intensity metrics). | We have addressed this by adding more detail in the Discussion. | Discussion softened to incorporate less persuasive language and reduce overinterpretation lines 523 - 526  We have also outlined why our interpretation is important in a new General considerations section lines 549 - 560 |
| 3  Specific comments: | The point in relation to the validity of the relative intensity construct should be elaborated in the discussion instead of the intro (line 161-166). The assumption made throughout the manuscript that exercising at the same relative percentage of HRmax (or any other maximum derived percentage) equals the same level of relative intensity between two individuals is incorrect. Although I might agree that using a percent approach is likely more accurate than using METs, these limitations should be well elaborated within the body of the discussion. | In relation to the assumption made throughout the manuscript, we don’t think we have made this assumption because we have addressed these limitations in the Introduction. That being said, we have moved that section to the Discussion and expanded upon this. | This has been amended on lines 542 - 546. |
| **4** | Line 457: this is a bit unclear. The “intensity” associated with a given MET is participant dependent. |  | Removed |
| **5** | Line 494-496: statements like this one make it sound as if this is the first piece of evidence demonstrating this discrepancy – which is not the case (again see Norton et al. discussing this issue quite few years ago). | This was never our intention, nor do we explicitly say that this is the first piece of research of its kind. However, we have addressed this for balance, by adding additional findings that align with this paper. | Norton 2010 and Ianetta 2021 references added, as well as ‘in line with previous findings’ line 523 |
| **6** | Line 502: Measures of relative intensities are already included, for example, in the ACSM guidelines. They are technically already adopted. The problem is that they are also limited. | We agree there are limitations with percentage of maximal intensity thresholds and have discussed this in the General considerations section in the Discussion. However, at the moment the technology to measure VT is simply not available in a wearable device accessible to the general public. So, our solution is a pragmatic one that is a balance between science and practice. | Changed from ‘adopted’ to ‘used’. We have addressed the limitations in relative intensity approaches highlighted in this paper in the General considerations section lines 540 - 560. |
| **7** | In general, the authors make strong statements in relation to the adoption of relative measures of intensity. But which one? %HRmax or %HRR? Or %VO2max? Not necessarily they coincide either (see recent work from Ferri Marini et al.). Then, how should the general public derive these measures? In my opinion, there are several physiological and methodological limitations with any of these propositions. I just would like the authors to recognize some of them to offer a more thorough view of the problem of exercise quantification and prescription (which, I agree with the authors, is an important one in our field). | Our preference is %HRR as the current best option for which relative measure to focus on because it’s the best compromise between using a relative measure that can be measured in practice. In the Discussion -> General considerations section we discuss future developments (see above comment). | We have addressed the limitations in relative intensity approaches highlighted in this paper in the General considerations section lines 540 - 560. |
| **Reviewer 2** |  |  |  |
| **2** | There is more to answering the criticism and suggestions of peers than just citing literature. Meta-analysis is a powerful tool to answer questions. However, unless an appropriate mechanistic context is provided, there is the risk to pose the wrong question as well as to misinterpret the results, which I think is the case for this manuscript. Appropriate physiological background needs to be in the paper. | We have addressed this by adding mechanistic context in the Discussion. | Added to General considerations in the Discussion. Line 573 - 584 |
| **4** | However, the correct approach to the matter is the physiology-based and data-driven revision of the fixed METs prescription scheme. This approach exists and has been published (Iannetta’s work as an example), it is valid across sexes and a very large age-range and it is clearly applicable outside laboratories. Moreover, a number of methods exist that allow an accurate estimate of absolute metabolic intensity based on the simple measure of HR in a variety of real-life contexts and populations (e.g. Wicks J, Pogliaghi S). These studies provide a broader context and support to your work and should be acknowledged and discussed. | We have referenced the Ianetta paper. However, the aim of this manuscript it not to revise fixed METs prescription. A fixed MET prescription scheme does not make relative intensity physical activity more accessible to the general public. We believe the best solution would be a fully individualised approach, that is not dependent on fixed percentages of maximal criteria, however this method is currently unavailable to the general public via a wearable device and thus we believe the best solution is to use %HRR as currently the most accessible relative measure. | References added line 523  We have explained the need for this development and current limitation in using a fixed percentage threshold to mediate exercise intensity boundaries line 540 - 560 |
| **5** | The limitation of using only walking, i.e. an activity that elicits at best a moderate intensity, is hardly suitable to establish a correspondence between methods of measurement of absolute and relative intensity. A wider range should be used. The implications of using a limited intensity range must be discussed by the authors. | Although we agree a wider spectrum of intensity of exercise modalities could be investigated, we chose to delimit our scope to walking because it is the most common form of physical activity. However, we acknowledge from a mathematical perspective comparing agreement over a wider range of intensities is warranted in the future. | We have addressed this in the General considerations section in the Discussion on Line 562 - 571 |
| **6** | More importantly, it is clear from the authors answer that in the majority of the studies included in the meta-analysis, actual METS were not directly measured but rather they were estimates. | In the Results section we outline the proportion of studies that directly measured METS, the proportion of studies that measured oxygen consumption during exercise but not resting oxygen consumption (hybrid) and estimated METs.  Although we haven’t attempted to say that measures of absolute intensity are not needed, this may have come across in the current manuscript. We have addressed this throughout the manuscript e.g. line 523 – 526 in the Discussion. We have softened our approach to relative intensity. We do not advocate dropping absolute intensity completely. | This has been described in the General considerations section line 663 - 565 |
| **7** | The authors need to discuss the implications of this on the lack of correspondence between absolute and relative exercise intensity. | The two papers used in the meta regression both used measured METs, and we have addressed this in the Discussion. | This has been described in the Results section line 399 – 403 and address in the Discussion. |
| **8** | The revised version of your manuscript clearly states that the low correspondence between absolute and relative intensity (as classified by the ACSM’s translation scheme) should lead us to focus on relative intensity only when prescribing exercise in the general population. Again, this may appear an easy fix but it is mechanistically incorrect. What your analysis confirms that the current mutual translation scheme between absolute and relative intensity should be substantially revised. | This is not the purpose of our manuscript. We believe there needs to be an increased focus on relative, individualised measures to better prescribe and monitor physical activity. We have addressed this in the general Discussion | Added to the general Discussion section line 540 - 560 |
| **9** | However, the importance of absolute intensity as an index of absolute energy expenditure, absolute heat production, absolute carbohydrates and fats oxidation, i.e. the “extensive” stress for the body that is generated by physical exertion should not be downplayed. This is just as relevant towards certain health related outcomes of the “exercise pill” as the “intensive” stress that is related to the disturbance of the intracellular homeostasis in myocytes, that in turn is related to relative intensity. Absolute and relative load are both “active principles” of the exercise pill that stress the body in a different way, causing different structural and functional adaptations over time (please see J Physiol 2017 May 1;595(9):2915-2930 and successive work from the same group for a general framework). This needs to be clearly presented in the manuscript. | We have added discussion on total energy expenditure and its relevance with absolute and relative intensity in the General considerations section. | We have added discussion on absolute and relative intensity exercise and its impact on energy expenditure in the General considerations section of the Discussion lines 573 - 584 |
